# Loss of neuromonitoring signal during bilateral thyroidectomy: no systematic change in operative strategy according to a survey of the French Association of Endocrine Surgeons (AFCE)

**DOI:** 10.1186/s12893-015-0082-5

**Published:** 2015-08-06

**Authors:** Lilly Khamsy, Paul E. Constanthin, Samira M. Sadowski, Frédéric Triponez

**Affiliations:** Faculty of Medicine, University of Geneva, Geneva, Switzerland; Thoracic and Endocrine Surgery, University Hospitals of Geneva, Geneva, Switzerland; Endocrine Oncology Branch, National Cancer Institute, National Institutes of Health, Bethesda, MD USA

**Keywords:** Intraoperative neuromonitoring, Bilateral thyroidectomy, Recurrent laryngeal nerve injury

## Abstract

**Background:**

Total thyroidectomy presents a risk of bilateral vocal cord paralysis, which can lead to compromised airway. Visual Recurrent Laryngeal Nerve (RLN) identification significantly decreases this risk of RLN lesion. Yet, an anatomically intact nerve is not always functional. Intraoperative neuromonitoring (IONM) allows to test in real time the function of the RLN. In case of loss of signal (LOS) on the first operated side, some authors recommend to stop the intervention. The purpose of this study was to characterize the operative strategy of the French-speaking surgeons in case of LOS on the first side in planned bilateral thyroidectomies.

**Methods:**

An online questionnaire was sent to the surgeons of the French Association of Endocrine Surgeons (AFCE).

**Results:**

We collected 69 responses (response rate: 42 %). Forty-six surgeons (66 %) used IONM. After a signal loss, 22 % (*N* = 10) stopped the operation in all cases, 24 % (*N* = 11) continued the operation in case of malignant disease and stopped in cases of benign disease, and 54 % (*N* = 25) continued the operation contralaterally.

**Conclusions:**

The majority of surgeons continued the operation contralaterally as originally planned despite a loss of IONM signal at the end of the first side.

## Background

The most feared complication following thyroid surgery is a lesion of the recurrent laryngeal nerve (RLN) [1]. Unilateral lesion usually causes hoarseness (with incapacity to produce explosive sounds) and an increased frequency of aspiration (food or liquids). Unilateral paralysis has no impact on patient survival, while paralysis of both vocal cords following a bilateral lesion of the RLN involves their prognosis. Indeed, paralyzed vocal cords can lead to an important risk of airway clogging [2], often making it necessary to perform transient or permanent tracheostomy. Despite of being rare (risk below 1 % [1–4]), this injury is serious, and if permanent, leads to long term disability.

Among many techniques developed to avoid injury to the RLN, the most effective and recognized one is dissection of the nerve throughout its retro-thyroid path [5]. However, an anatomically intact RLN is not necessarily functional. Intraoperative neuromonitoring (IONM), based on electrophysiology, tries to compensate for this discrepancy [6] by checking in real time the functional integrity of the loop between the RLN and the laryngeal muscles it innervates. The nerve is electrically stimulated using a stylus. This stimulation results in the contraction of the voice muscles whose depolarization is measured by the electrodes present on the endotracheal tube. The presence of a muscle response to the nerve stimulation is transmitted to the surgeon by an audible and visual EMG signal on the monitor, thus confirming the functional integrity of the RLN. The presence of a good EMG signal by stimulation of the vagus nerve at the end of dissection is known to confirm the absence of laryngeal paralysis with an accuracy of more than 99 % using strict criteria [1]. Some authors have begun to define normal values for this EMG signal [7, 8]. A loss of signal (LOS) can be observed in various situations; when the stylus touches another structure instead of the RLN or when the patient is under myorelaxant medication, thereby preventing the contraction of the voice muscles, or when there is a lesion of RLN. Another confusing factor can be the preoperative state of the vocal cords [9]. The use of algorithms such as those described by the International Neural Monitoring Study Group reduce signal losses that do not represent nerve injuries [10].

Since it is difficult to differentiate between situations that lead to LOS, some centers, such as the University Hospitals of Geneva (HUG), created an in-hospital use of IONM recommendations [11]: A LOS after dissection of the first side and after exclusion of technical problems is considered to be a probable injury to the RLN and the contra-lateral side is then not approached [12]. Therefore, an operation is interrupted after the first side in these situations. The patient is then followed by laryngoscopic control to assess the laryngeal impairment, and according to the recovery or not of a good RLN function, another treatment option is sometimes considered for treatment of the opposite side (conservative follow-up of goiter, radioactive iodine for the treatment of hyperthyroid patients for instances) [12].

Although the use of IONM could possibly decrease the rate of transient postoperative speech modifications [13], it has not demonstrated real benefits in reducing intraoperative lesions of RLN [6]. In a recent study [14], 60 % of patients who had LOS showed no laryngeal palsy (positive predictive value 40 %). Therefore, this technique is subject to controversy within the community of surgeons; some arguing that it is inefficient and questioning it’s reliability.

According to a study conducted in Germany by Dralle et al. [15], 93.5 % of surgeons who use IONM interrupt the scheduled bilateral thyroidectomy after LOS on the first side in order to reduce the risk of bilateral paralysis of the RLN to 0 % [12]. Randolph et al. recommend to standardize the use and impact of IONM on the surgical strategy and encourage surgeons who face a LOS at the end of the first lobectomy to reassess the utility of further surgery and possibly to delay the contralateral treatment [10].

On the other hand, Sitges-Serra et al. do not advocate stopping the operation when LOS occurs during the first side of surgery. According to them, continuing the operation of the second side does not increase the risk of bilateral RLN injury. In their study, only 1 patient out of 16 suffered transient unilateral RLN paralysis. In addition, the signal from the first side recovered at the end of the operation in up to 90 % of cases. Therefore, they suggest that bilateral thyroidectomy can be performed without risk of bilateral laryngeal paralysis despite LOS upon the first side [16].

Faced with these divergent views on the appropriate response to LOS, we inquired about the protocol applied by the French surgeons. Does LOS during bilateral thyroidectomy induce a change in their operative strategy?

## Methods

Based on a literature review regarding intermittent IONM of RLN we created an online questionnaire, entailing 20 questions of closed type, single or multiple-choice and open field responses (see appendix 1, the original questionnaire in French and appendix 2, the questionnaire translated in English). This questionnaire did not require submission and approval from the Ethic Committee.

We sent the questionnaire to 164 members of the French speaking Association of Endocrine Surgeons (Association Francophone de Chirurgie Endocrinienne, AFCE). Data were collected over a 6 months period (with a second send-out and a reminder). We closed the survey in March 2013.

We analysed the data using the statistical software SPSS ® version 20.0.0, using the Chi-square test and Fisher’s exact test for categorical and continuous variables, respectively. Statistical significance was set at the 0.05 level.

Anonymity was preserved throughout the study.

## Results

We received 69 responses from 65 different institutions out of 164 e-mails (42 %), incoming from 11 countries (50 from France, 72 %, 6 from Belgium, two from Switzerland, two from Italy, two from Spain, two from Canada, one from Algeria, one from Germany, one from England, one from Lebanon and one from Mexico). The mean time to complete the questionnaire was 5 min (range from 2’45 – 13’20 min), and 66 (96 %) of the questionnaires were fully completed.

Most of the participants (80 %) were 40 – 65 year old (Table 1) Their basic training was in general surgery (*N* = 54, 78 %), ENT (*n* = 7, 10 %), thoracic surgery (*N* = 6, 9 %) or other specialties (*N* = 2, 3 %). During the study period, most surgeons practiced at university hospitals (*N* = 35, 53 %), some worked in private non-university hospitals (*N* = 19, 29 %), non-university public hospitals (*N* = 7, 11 %), in non-profit institutions (*N* = 2, 3 %) or other institutions (*N* = 3, 4 %).

**Table 1:** Age of participants

Age range	Number/percentage
<30	1/1.4 %
30–39	7/10.1 %
40–49	28/40.6 %
50–65	27/39.1 %
>65	6/8.7 %

A majority of surgeons (75 %) had performed more than 500 thyroidectomies at the date of the survey. (Table 2) They performed on average over 200 thyroidectomies per year (*N* = 25, 35 %), between 50 and 200 per year (*N* = 35, 52 %) and between 0 and 50 per year (*N* = 9, 13 %). Years of experience for thyroidectomies were on average 16 years (+ / - 9 years, range 1–37 years).

**Table 2:** Total number of thyroidectomies that had been performed by each participant at the date of the survey

Number of thyroidectomies performed	Number/percentage
<100	1/1.5 %
100–500	16/23.5 %
500–1000	10/14.7 %
>1000	41/60.3 %

In our cohort, a minority of surgeons did not use the IONM (*N* = 24, 34 %), some never used it (*N* = 20, 28 %), while others were in the process of acquiring equipment or training (*N* = 4, 6 %).

Among the IONM users (*N* = 46, 66 %), some declared using the device on a daily basis (*N* = 24, 34 %) and others only occasionally (*N* = 22, 32 %). Ten (22 %) participants had less than one year of experience using IONM, seventeen (37 %) between 1–3 years of experience of use, nine between 3–5 years (20 %) and ten more than 5 years (19 %). They used it during all operations (22 %), during revision surgery (21 %) or selectively in some indications (57 %, oncological pathologies, bilateral thyroidectomy, Graves’ disease, retrosternal goiters among others).

**Fig. 1:**
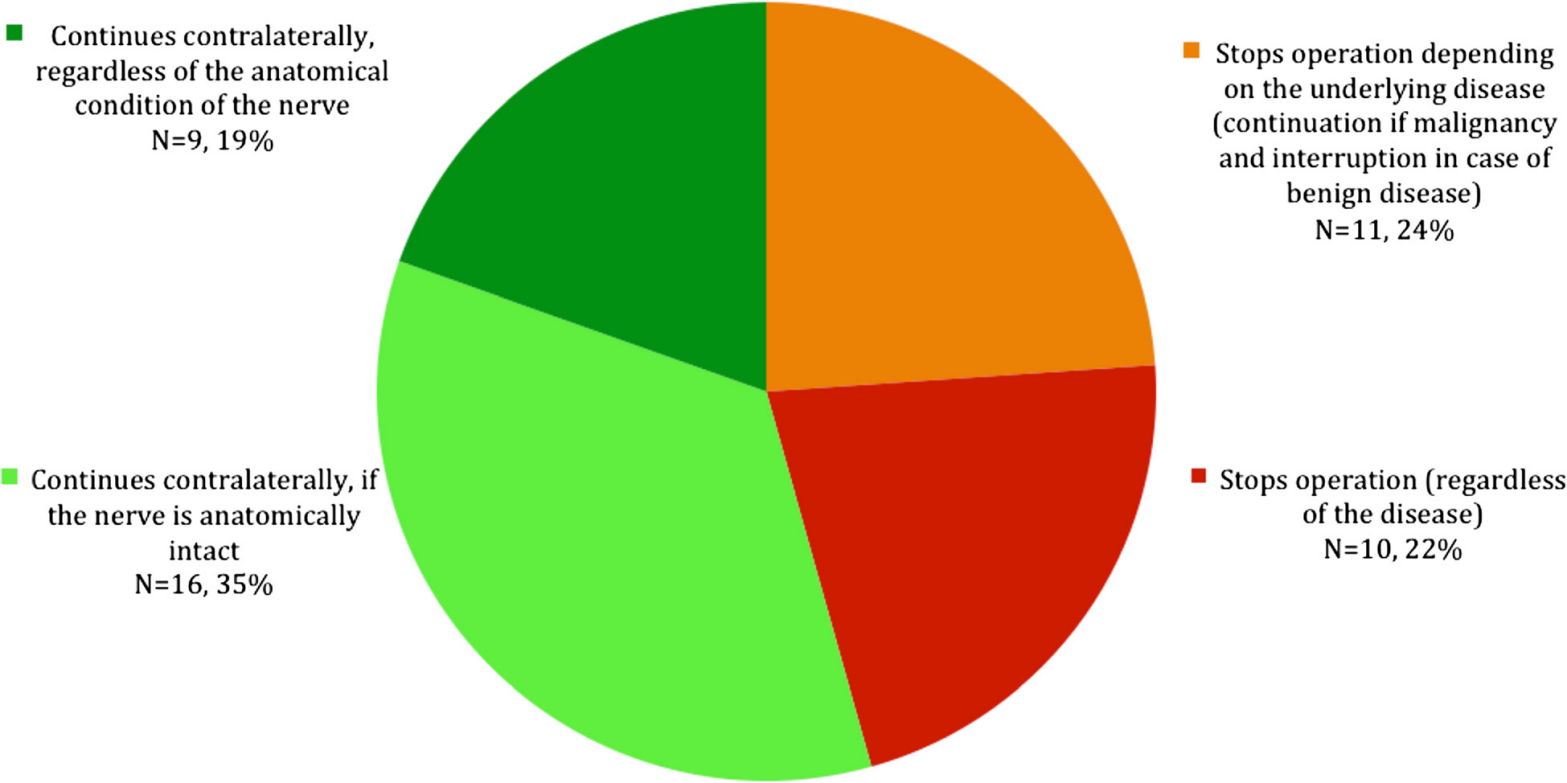
Reaction following losss of signal during an initially bilateral-scheduled thyroid operation (*N* = 46)

Upon losing the EMG signal during an initially bilateral-scheduled operation, 19 % of surgeons (*N* = 9) carried on to the contralateral side, regardless of the anatomical condition of the nerve. 35 % of the surgeons (*N* = 16) continued their procedure only if the nerve was anatomically intact, overall 22 % (*N* = 9) stopped the operation (regardless of the disease), and 24 % (*N* = 11) stopped the operation depending on the underlying disease (continuation if malignancy and interruption in case of benign disease) (Fig. 1).

### Influence of surgeon’s background on operative strategy

Neither the age, the number of annually performed thyroidectomies or the type of hospital significantly influenced the surgical strategy. In contrast, surgeons who had more experience (>1000 thyroidectomies) were significantly more likely to pursue the operation, compared to less experienced surgeons (<1000 thyroidectomy) (*p* = 0.022). Similarly, surgeons who had been using IONM for more than a year pursued more often than new IONM users (*p* = 0.042) (Table 3).

**Table 3:** Influence of surgeons background on operative strategy

Characteristics	*P* value
Age of surgeon	0.40
Number of annually performed thyroidectomies	0.089
Type of hospital (university, public non university, private, etc.…)	0.385
Surgeon’s experience in thyroidectomy (>1000 vs < 1000 thyroidectomies performed)	0.022
Experience with IONM (>1 year vs < 1 year of IONM use)	0.042

Finally, among surgeons who did not use the IONM (*N* = 23, 33 %), and faced an accidental RLN lesion, most (*N* = 11, 48 %) carried on with the operation on the contralateral side, regardless of the underlying disease, whereas 43 % proceeded only when surgery was performed for malignant cases (*N* = 10). Further, 9 % of IONM-non users systematically interrupted surgery when recognizing an iatrogenic RLN lesion (*N* = 2).

## Discussion

Bilateral lesion of the RLN during thyroid surgery remains a serious and feared complication, owing to its impact on the prognosis and on patient’s quality of life. Functional impairment of the RLN is not always visible macroscopically. Therefore, IONM has been proposed to facilitate the identification of functionally-relevant nerve damage. Despite the lack of evidence to support a benefit of IONM over the current gold-standard retro-thyroid RLN dissection, many surgeons have adopted IONM in their daily practice.

We observe that a minority of surgeons in our cohort applies the protocol recommended by most experts [10]. In most cases, LOS on the first side does not lead to an interruption of the operation. In our cohort, the majority of surgeons who use IONM continues the operation on the contralateral side despite LOS. Some surgeons base their decision upon macroscopic appearance of the nerve, some on the disease malignancy and some continue the operation in all cases. Their experience seems to outweigh the information brought by IONM. Indeed, only a minority of 22 % IONM users systematically adapts their operating strategy following LOS.

The macroscopic aspect of the RLN seems superior to the functional information transmitted by IONM, as suggested by the the operative strategy of surgeons who proceed after LOS when the RLN is anatomically intact. However, according to a study by Goretzki et al., the stress following the loss of the first signal may increase the risk of paralysis on the other side [17].

We observed that the reaction to the LOS was influenced by the experience of surgeons: surgeons who performed more thyroidectomies in their careers tended to continue while less experienced tended to stop the operation. Similarly, surgeons who used IONM for less than a year often adapted the operative strategy and halted the procedure. In contrast, the surgeon’s age or annual volume of thyroidectomies did not significantly affect the operative strategy. Thus, despite a LOS during IONM, the more experienced surgeons rarely stopped a planned bilateral thyroidectomy, similar as the highly experienced team of Sitges-Serra et al. [16]. It is likely that the experienced surgeon estimates the bilateral recurrent laryngeal nerve palsy to be a very rare complication.

However, it is crucial to avoid bilateral laryngeal paralysis. Although Barczynski et al. suggest a decrease in transient voice changes postoperatively using IONM [13], most studies have failed to demonstrate a statistically significant decrease in the risk of laryngeal paralysis using IONM [18]. This complication is so rare, that the statistical power of the studies might be too low to obtain significant results. According to Dralle et al. the risk of laryngeal paralysis is lower than 0.1–1 % when a good EMG signal is present [1]; adding IONM to the careful dissection of the RLN, one would approach zero risk. In contrast, the positive predictive value when a signal is lost, is about 40 %, and does not always represent a lesion of the RLN [14]. In addition, the absence of signal does not predict the reversibility or irreversibility of a laryngeal lesion. Thus, it is possible that surgeons do not rely on this technique to test nerve function, but use it essentially for its secondary use, to identify the RLN.

Our results raise the question of the benefit of IONM, as the information provided on nerve function through LOS does not influence the surgical strategy. This could lead the surgeons to reconsider the usefulness of using IONM or their reaction to LOS in order to take advantage of the information provided. With the recent release of international recommendations [10], it is possible that the surgical strategy in response to LOS will be homogenized and the role of IONM in bilateral thyroidectomy clarified. Is the device used as an operating aid or as a diagnostic tool for possible nerve damage? Moreover, future studies should determine how a two-staged operation strategy affects the health costs [19], the medico-legal, psychological and ethical aspects [20, 21].

We do not know from this study whether responders didn’t modify their operative strategy because they weren’t yet aware of the international recommendations or whether they purposely didn’t follow these recommendations based on different personal experiences. The introduction of neuromonitoring of the RLN in France took longer than in other countries (like Germany for instance) and it is very possible that the responders of this survey were “at the beginning” of their neuromonitoring experience (as compared to many German surgeons for instance) and that they are still uncertain about the strategy to adopt in case of a LOS after dissecting the first side. The fact that only 39 % of the surgeons used IONM for more than three years in this survey, suggests that this hypothesis is very likely. It would be interesting to see whether the answers from the same surgeons will be different in a few years. On the other hand, experienced authors continue the surgery after LOS and from 16 LOS in 290 patients, they demonstrated that 15 of them recovered after completion of the second side, after a mean of 20.2 min. They did not experience any LOS on the contralateral side in their study [16]. This study is interesting but does not correspond to the generally published series reporting a minimum of 50 % rate of RLN palsy after LOS, which is also our experience. Whether technical aspects differed in their practices, is difficult to extrapolate from their study. In our opinion, the safety of the patients comes first and any intra-operative suggestion that the first dissected RLN could be non-functioning should lead to an interruption of the surgery; to date, other than transection of the RLN, LOS during IONM is the most reliable tool to suggest a non-function of the RLN.

There are shortcomings to our study. First, the limited response-rate (42 %) is a drawback to the generalizability of our findings. However, some members of the French speaking Association of Endocrine Surgeons to whom the questionnaires were sent are not surgeons (endocrinologists, pathologists, oncologists, radiologists), and some members are retired. Moreover, it seems that many surgeons not using IONM did not answer the questionnaire because they thought it was only meant for the IONM users. Therefore, we believe that the “true” response rate from actively practicing surgeons is probably higher than the 42 % “crude rate”. Second, previous lines of evidence have shown that a majority of thyroidectomies involve surgeons with limited case load (<50 thyroidectomies per year) [22, 23]. Thus, one could wonder whether our cohort, who included a noticeable proportion of experienced thyroid surgeons, accurately reflects daily practice in other settings. Third, this questionnaire was set up in 2012 at a time when continuous monitoring was at its very early phase [24] and therefore this study does not explore the current practice of surgeons using continuous monitoring. Fourth, we did not investigate what criteria each surgeon used to define LOS because the aim of the study was to investigate the strategy adopted after experiencing a LOS, whatever criteria each individual surgeon used to define LOS.

## Conclusions

The reaction after EMG signal loss during IONM in bilateral thyroidectomy is not unanimous in French speaking Endocrine Surgeons. Some surgeons ignore the information given on the functional state of the RLN by IONM and base their judgement on the anatomical integrity of the nerve to decide whether to continue the operation or not.

The recent publication of guidelines could harmonize practices in this area and it would be interesting to follow their evolution in the coming years.

## Abbreviations

(IONM): Intraoperative neuromonitoring
(RLN): Recurrent laryngeal nerve
(LOS): Loss of signal
